# Increase in Beta Power Reflects Attentional Top-Down Modulation After Psychosocial Stress Induction

**DOI:** 10.3389/fnhum.2021.630813

**Published:** 2021-03-23

**Authors:** Ismael Palacios-García, Jaime Silva, Mario Villena-González, Germán Campos-Arteaga, Claudio Artigas-Vergara, Nicolas Luarte, Eugenio Rodríguez, Conrado A. Bosman

**Affiliations:** ^1^Laboratorio de Neurodinámica Básica y Aplicada, Escuela de Psicología, Pontificia Universidad Católica de Chile, Santiago, Chile; ^2^Centro de Estudios en Neurociencia Humana y Neuropsicología, Facultad de Psicología, Universidad Diego Portales, Santiago, Chile; ^3^Centro de Apego y Regulación Emocional, Facultad de Psicología, Universidad del Desarrollo, Santiago, Chile; ^4^Clínica Alemana de Santiago, Santiago, Chile; ^5^Department of Physiology, Faculty of Biological Sciences, Pontificia Universidad Católica de Chile, Santiago, Chile; ^6^Cognitive and Systems Neuroscience Group, Swammerdam Institute for Life Sciences, Center for Neuroscience, University of Amsterdam, Amsterdam, Netherlands; ^7^Research Priority Program Brain and Cognition, University of Amsterdam, Amsterdam, Netherlands

**Keywords:** anxiety, heart rate (HR), attentional control, beta band frequency, psychosocial stress

## Abstract

Selective attention depends on goal-directed and stimulus-driven modulatory factors, each relayed by different brain rhythms. Under certain circumstances, stress-related states can change the balance between goal-directed and stimulus-driven factors. However, the neuronal mechanisms underlying these changes remain unclear. In this study, we explored how psychosocial stress can modulate brain rhythms during an attentional task and a task-free period. We recorded the EEG and ECG activity of 42 healthy participants subjected to either the Trier Social Stress Test (TSST), a controlled procedure to induce stress, or a comparable control protocol (same physical and cognitive effort but without the stress component), flanked by an attentional task, a 90 s of task-free period and a state of anxiety questionnaire. We observed that psychosocial stress induced an increase in heart rate (HR), self-reported anxiety, and alpha power synchronization. Also, psychosocial stress evoked a relative beta power increase during correct trials of the attentional task, which correlates positively with anxiety and heart rate increase, and inversely with attentional accuracy. These results suggest that psychosocial stress affects performance by redirecting attentional resources toward internal threat-related thoughts. An increment of endogenous top-down modulation reflected an increased beta-band activity that may serve as a compensatory mechanism to redirect attentional resources toward the ongoing task. The data obtained here may contribute to designing new ways of clinical management of the human stress response in the future and could help to minimize the damaging effects of persistent stressful experiences.

## Introduction

Attention is defined as the ability to select and process sensory stimuli, thoughts, and relevant actions while ignoring irrelevant distractors from a complex environment. Attention usually shifts between endogenous top-down content (for example, goals and expectations) and exogenous bottom-up influences (for example, sensory-driven stimulation; Corbetta and Shulman, 2002). The ability to allocate attentional resources according to the context originates from the activity of an extensive brain network, including the intraparietal sulcus/superior parietal lobe, the dorsal frontal cortex, the dorsolateral prefrontal cortex, and subcortical structures (Corbetta and Shulman, 2002; Silver and Kastner, 2009; Petersen and Posner, 2012; Morillas-Romero et al., 2015; Fiebelkorn and Kastner, 2020).

Psychosocial stress is considered an important modulator of selective attention (Arnsten, 2009; Qin et al., 2009; Veer et al., 2011; Hermans et al., 2014; Marshall et al., 2016; Marshall and Cooper, 2017; Van Oort et al., 2017). Usually, a psychosocial stress response is activated by unpredictable and uncontrollable social situations, in which the subject anticipates the psychological consequences of social behavior. It induces physiological and psychological responses such as increased heart rate (HR) and anxiety levels. This stress also influences the allocation of attentional resources and modulates the activity of the attentional network. For example, students reporting high levels of perceived stress due to academic load exhibit disrupted attentional control and decreased functional connectivity between the dorsolateral prefrontal and posterior parietal cortex (Liston et al., 2009). In experimental paradigms used to induce psychosocial stress, participants exposed to an evaluated interview followed by an arithmetic task showed strengthened connectivity between the amygdala and brain regions such as the locus coeruleus, dorsal anterior cingulate cortex, and anterior insula (Van Marle et al., 2010; Veer et al., 2011; Hermans et al., 2014 ). These studies suggest that a decrease in the prefrontal cortex activation induces higher vigilance levels, probably due to a reallocation of the attentional control (Roelofs et al., 2007; Arnsten, 2009; Hermans et al., 2014). However, despite these advances, we do not completely understand how psychosocial stress modulates the functional dynamics of the attentional networks.

The activity of brain networks can be characterized through the study of the power and phase relationships across neuronal rhythms (Engel et al., 2001; Engel and Singer, 2001; Fries, 2005; Palva et al., 2005; Jensen et al., 2007; Bosman et al., 2014; Pesaran et al., 2018). Several studies have shown the modulatory effects of stress-derived states, such as anxiety, over brain oscillations (Knyazev et al., 2005; Lewis et al., 2007). For example, increased anxiety correlates with delta (4–6 Hz) and beta (13–29 Hz) oscillations in response to speech anticipation (Miskovic et al., 2010). Similarly, an alpha frequency band (8–12 Hz) power increase is specifically associated with individuals with high-anxiety traits during resting state and attentional tasks (Knyazev et al., 2004, 2005, 2006), and with attentional reallocation toward inner thoughts and mind-wandering (Cooper et al., 2003; Klimesch, 2012; Villena-González et al., 2017). Nevertheless, it remains unclear how psychosocial stress modulates the activity of brain rhythms during attention.

Recently, it has been shown that top-down attentional modulation correlates with an increase in beta band power and phase synchronization between brain areas (van Kerkoerle et al., 2014; Bastos et al., 2015; Michalareas et al., 2016), whereas stimulus-dependent bottom-up attention correlates with an increased gamma band (30–90 Hz) power and phase synchronization (Buschman and Miller, 2007; Siegel et al., 2008; Gregoriou et al., 2009; Bosman et al., 2012; Grothe et al., 2012; Buschman and Kastner, 2015) during selective attention. Based on these findings, we hypothesized that psychosocial stress might increase the self-reported state of anxiety, triggering a shift from exogenous to endogenous cues during an attentional task. This shift might be reflected in a decrease in the accuracy of behavioral responses and an increase in the oscillatory signatures reflecting top-down responses (increases in the alpha and beta frequency band activity and a decrease in the activity of the gamma frequency band).

## Materials and Methods

### Participants

Forty-nine male subjects were recruited and randomly assigned to the psychosocial stress induced (*n* = 24) or control (*n* = 25) groups. All participants had normal or corrected-to-normal vision and reported no color vision deficiencies. The participants had no history of drug abuse or neurological or psychiatric conditions. Seven of these participants (four controls) were excluded because they failed to follow instructions during the experiment or data acquisition problems. A final sample size of 42 non-medicated male volunteers (mean age ± SD, 25 ± 3.8 years) was recorded between 12.00 and 14.30 h. All participants signed written informed consent before the study, following the Bioethics Committee of the Faculty of Medicine guidelines at the *Pontificia Universidad Católica de Chile*, which approved the research protocol.

### Psychosocial Stress Induction

**Figure 1:**
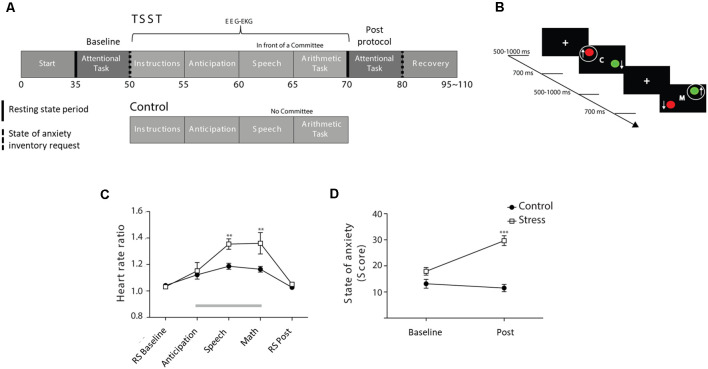
Physiological and subjective stress markers. **(A)** Schematic diagram of the experimental setup. Continuous and discontinuous black vertical lines indicate resting-state periods and state of anxiety inventory requests, respectively. The numbers below the diagram indicate the approx. time in minutes of the respective experimental session. **(B)** Attentional paradigm: an initial central fixation cross appears at a random interval from 0.5 to 1 s, followed by 0.7 s of the trial epoch, in which the participant has to choose between the color or motion of two circles dependent if a central word is a ‘C’ of color or an ‘M’ of motion. During the first two blocks of the task, the participant has to choose the green and upward circles. During the last two blocks, subjects have to switch their attention to the red and downward circles. **(C)** During each period of the experimental procedure for the Trier Social Stress Test (TSST) and Control group, the heart rate (HR) ratio is shown. Gray bar depicts the moment of the the TSST or control protocol, **p < 0.01. **(D)** State of anxiety score during baseline and after the TSST or control protocol, ***p < 0.001. Data plots are presented as the mean ± SEM.

To induce psychosocial stress under controlled conditions, we used an EEG-adapted version of the Trier Social Stress Test (TSST) protocol, following the guidelines of Kirschbaum et al. (1993; Figure 1A). The protocol consists of a simulated job interview in which the participants must prepare a public speech about their attributes for a fictional job (anticipatory phase, 5 min) to subsequently deliver the speech in front of three referees in an expressionless but severe attitude (speech phase, 5 min). This phase is followed by a mental arithmetic task consisting of counting backward from 1,033 to 0 in steps of 13 (arithmetic phase, 5 min).

The control protocol included the same procedure as previously described, but including only one of the experimenters with the participant displaying a good mood and friendly attitude. The same phases were induced but without the psychosocial stress component. After the protocol, the participants were informed that their performance was not evaluated.

### Attentional Task

The stress-induced attention modulation was evaluated using an adaptation of a task-switching paradigm previously described by Liston et al. (2006; Figure 1B). In this paradigm, two circles, each subtending 4.6° of visual angle and equidistant to the monitor center, were presented for 0.7 s. Each circle was colored red or green and moved upward or downward. At the fixation point, there was a letter “M” for movement or “C” for color at the center of the screen. Participants were asked to choose either the green circle when the letter “C” appeared on the screen or the upward circle when the letter “M.” After two blocks, participants were instructed to choose red or downward circles when the “C” or “M,” respectively, appeared on the screen. Each trial began with a central white fixation cross of variable duration (0.5–1 s). The full trial involved central fixation followed by 0.7 s of colored and mobile circles (Figure 1B). Participants were trained with three blocks of 12 trials, corresponding to “*only-color*,” “*only-movement*,” and *color/movement* discrimination conditions. The experiment involved four blocks of 64 trials separated by a 1-min duration rest between blocks.

We recorded three different estimators of accuracy: the number of correct trials, the maximal number of consecutive failed trials (error or omission), and the number of episodes during the task with more than two successive failed trials (error or omission). The reaction times were recorded on a trial-by-trial basis using the software for stimuli presentation control, Psychopy (Peirce, 2008). The resulting values for each measurement were calculated as the difference between the post-protocol task scores and the baseline.

### Experimental Procedures

Both the control and stress-induced groups underwent the following experimental procedure: (1) 90 s of task-free EEG recording (baseline resting-state); (2) EEG recording during the performance of the attentional task (baseline attentional task); (3) psychosocial stress induction or control protocol; (4) 90 s of task-free EEG recording (post-resting-state); and (5) EEG recording while subjects repeated the attentional task (post-induction attentional task). After the completion of the attentional tasks, subjects were asked to fill the state of anxiety inventory (Spielberger, 2010; Figure 1A). During the task-free recording, participants were instructed to look at a fixation mark on the screen. This period will be referred to in the manuscript as “resting-state.” All the measurements performed before the TSST/control protocol will be designated throughout the manuscript as “baseline,” while the measurements performed after the protocols will be referred to as “post.”

### Physiological and Psychological Responses

ECG activity was monitored during all sessions using two external electrodes (BioSemi ActiveTwo^®^) positioned below the left clavicle and over the left hip. We obtained five samples of 90 s during the two resting-state periods (RS baseline and RS post) and at the beginning of the control/TSST tasks (Anticipation-Speech-Math) and used them to calculate the HR ratio. A normalized HR ratio was calculated by dividing each of the five samples by the lower HR. The HR was obtained and calculated using Kubios software (Tarvainen et al., 2014) and custom-made MATLAB scripts, respectively.

The evaluation of baseline subjective stress levels and anxiety traits was done using the perceived stress (Cohen et al., 1983) and trait anxiety (Spielberger, 2010) scales. The state of anxiety scale (Spielberger, 2010) was used to evaluate the psychological experience associated with the experimental design. A unique value was obtained by subtracting the state of anxiety post-treatment from the baseline.

### EEG Recording and Pre-processing

The EEG data were obtained using 64 electrodes (Biosemi^®^ ActiveTwo) arranged according to the international 10/20 extended system. Eye movements were monitored using four external electrodes. The horizontal EOG was recorded bipolarly from the outer canthi of both eyes, and the vertical EOG was recorded from above and below the participant’s right eye. Two electrodes were placed over the right and left mastoids for use as an offline reference. EEG, ECG, and EOG data were collected at a sampling frequency of 2,048 Hz. After the recordings, the data were downsampled to 1,024 Hz and re-referenced to mastoids using the MATLAB toolbox EEGLAB v7.1.7.18b (Delorme and Makeig, 2004).

### Data Analysis

The recording datasets from both the resting state and task periods were initially processed through a band-pass filter between 0.1 and 80 Hz using a 2nd order infinite impulse response (IIR) Butterworth filter implemented in the EEGLAB toolbox (Delorme and Makeig, 2004), followed by a discrete Fourier transform (DFT) filter between 48 and 52 Hz, in steps of 0.01 Hz, using 10 s of mirror padding. Subsequently, EEG artifacts were rejected by visual inspection and subjected to independent component analysis (ICA). Blink and cardiac artifacts were rejected using an ICA algorithm, implemented in EEGLAB (Delorme and Makeig, 2004), and a previously described procedure to eliminate cardiac artifacts from the EEG (Jung et al., 1998). Each resting-state 90 s period was divided into 0.5 s epochs. The task data were segmented between 0.5 s before the stimulus onset and 1 s after stimulus presentation. Artifact-free datasets were filtered and analyzed using the MATLAB FieldTrip toolbox (Oostenveld et al., 2011).

The power spectrum was obtained after applying a Hanning-taper Fourier Transform to the filtered data. The time-frequency power amplitude across trials was obtained using a Fourier analysis with a sliding window of 0.3 s in steps of 30 ms and normalized into a Z-score relative to the baseline (from 0.35 s previous to the stimulus presentation until the stimulus onset). Connectivity analyses were performed on the resting period data using the weighted phase-lag index (WPLI; Vinck et al., 2011), a measure of phase synchronization with reduced sensitivity for volume conduction artifacts and improved statistical power, to detect transient phase synchronization events. These analyses were performed between the frontal and parietal 9-electrode clusters centered on the electrodes Fz (AF3, AFz, AF4, F1, Fz, F2, FC1, FCz, FC2) and Pz (Cp1, CPz, CP2, P1, Pz, P2, PO3, POz, PO4), respectively.

### Statistical Analysis

The effects of the psychosocial stress elicited by TSST over HR and state of anxiety were evaluated with a two-way repeated measure analysis of variance (ANOVA), using a Bonferroni post-test correction. The relationship between the different variables was calculated using Pearson’s correlation with the software GraphPad Prism (GraphPad Software, San Diego, CA, USA).

#### Permutation Test and Multiple Comparison Correction

The power amplitude and resting-state connectivity differences were statistically assessed using a non-parametric permutation test corrected by multiple comparisons across all frequencies between 8 and 80 Hz (Nichols and Holmes, 2002; Maris and Oostenveld, 2007; Bosman et al., 2012). The time-frequency charts obtained during the attentional task were compared between groups using a bin-by-bin permutation test (2^21^ repetitions) corrected by multiple comparisons. Briefly, the *T*-statistic between groups for every frequency bin (power spectrum and resting-state connectivity) was calculated. Next, 1,000 randomizations were performed, in which the epochs from both groups were permuted without replacement. The maximum and minimum values from the *t*-statistics were extracted from which obtained two random distributions of maximal and minimal differences were obtained. Finally, the experimentally observed *t*-statistics were compared with the maximal and minimal distributions. If differences were smaller than the 2.5th percentile of the minimal distribution or larger than the 97.5th percentile of the maximal distribution, they were considered significant at *p* < 0.05. This corresponds to a two-sided test with multiple comparison corrections across frequencies (Maris and Oostenveld, 2007).

For the time-frequency statistic tests, a bin-per-bin permutation was performed, and the resulting *t*-statistic for each bin of the time-frequency analyses was obtained. Multiple comparison correction was performed using the cluster method (Maris and Oostenveld, 2007). In this case, the largest significant time-frequency cluster for each permutation was identified with *p* < 0.05. Then, the cluster’s threshold size was adjusted to obtain the 1st percentile value from the cluster distribution with *p* < 0.05. Clusters with values below the threshold size were eliminated.

#### Logistic Regression Models

Several logistic regression models were fitted to evaluate the relationship between power and connectivity dynamics and the subjects’ behavioral states. Three binary classifiers were fitted using R software (R Core Team, 2020), which used either the beta band, gamma band, or only the intercept as covariates.

To assess how informative each model was about the experimental protocols, we used the likelihood-ratio test to compare both the beta- and gamma-band models against our “null” model consisting of the intercept only. This procedure allowed us to test the significance of each covariate and to determine the best model. Given that our estimates were derived from a relatively small sample size, logistic regression with few covariates (such as our models) should comply with minimal sample size requirements by the event per variable guideline (for an example, see Bujang et al., 2018).

To quantify the models’ classification accuracy, we performed a repeated k-fold cross-validation, using 10 repetitions and 10-folds (Rodriguez et al., 2009). This procedure allows the testing of the model’s predictions in “unseen” data accounting for over or under-fitting. The original data is split into “k-folds,” then models are fitted in k-1 of such folds and successively tested in the remaining fold. This procedure is repeated until every fold is used for data testing at least 10 times. After every iteration, a confusion matrix is returned, containing the predicted (experimental condition) and true classes in each row and column, respectively. These matrices are averaged through all iterations to obtain a model accuracy (correct classifications of overall classifications made). The area under the receiver characteristic curve (AUROC) indicates the model’s true positive and negative prediction rates over a range of thresholds, providing an estimation of model performance over possible thresholds (Steyerberg et al., 2001). Finally, to test the model accuracy, we computed a Kappa statistic, which effectively compares the observed accuracy with the expected accuracy (given by the class ratio).

## Results

### Psychosocial Stress Induces Physiological and Subjective Responses

First, we evaluated the HR changes and state of anxiety as outcomes of the stress response. We did not observe differences in the heart frequency between groups during resting-state periods (Figure 1C, resting-state baseline, resting-state post, Bonferroni post-test; *p* > 0.05). In contrast, we observed an increase in HR during the execution of the speech and arithmetic phases during the TSST and control protocols (Figure 1C, gray bar; Time effect *F*_(4,160)_ = 21.93; *p* < 0.001). This overall increase in HR was significantly stronger during the TSST (Figure 1C, Group × Time interaction effect *F*_(4, 160)_ = 3.645; *p* < 0.01) during the arithmetic and speech phases of the task, but not during the anticipation period (Figure 1C, Bonferroni post-test; **p < 0.01, ***p < 0.001).

We also assessed the state of anxiety after the presentation of each attentional task (Figure 1A, discontinuous black lines). We observed a substantial increase in anxiety after the execution of TSST, which was absent in the participants who underwent the control protocol (Figure 1D, Group × Time interaction effect, *F*_(1,40)_ = 38.04; *p* < 0.001). Importantly, this effect was elicited only after TSST (Bonferroni post-test; *p* < 0.001), indicating that the TSST protocol specifically triggered a stress-related increase in the state of anxiety levels.

### Psychosocial Stress Decreases the Improvement of the Behavioral Performance During the Attentional Task

We found that participants in both groups improved their scores on the attentional task compared with the baseline performance. However, this increment was significantly higher in the control group than in those participants experiencing stress induction (data not shown, two-tails Student’s *t*-test, Control: 11.29 ± 1.837 correct responses, Stress: 2.905 ± 2.318 correct responses, *t* = 2.834, *p* < 0.01).

Next, we correlated this performance impairment with the HR responses and self-reported state of anxiety. We observed that the self-reported state of anxiety, but not the physiological HR response, was directly correlated with performance in the attentional task (Table 1). Furthermore, we found an inverse relationship between the self-reported state of anxiety and the total number of correct trials, and a positive correlation between the self-reported state of anxiety and the maximal number of consecutive failed trials (Table 1). The control and stress-induced groups showed faster reaction times after the execution of the protocol despite changes in anxiety levels (Supplementary Figure 1D). Additionally, we found that the average number of episodes for two or more consecutive failures decreased similarly after both the control and TSST protocols (Supplementary Figure 1C). Finally, we found an increased number of maximal consecutive failed trials after psychosocial stress induction (Supplementary Figure 1B). These findings suggest that participants in both groups suffered a similar number of attentional lags across the task, but those in the stress-induced group were longer in duration, possibly affecting the overall improvement during the task performance.

**Table 1:** Correlation between stress outcomes and attentional performance.

		Max fails	Number of events	Reaction time	Anxiety state	Heart rate
Corrects
*R*	1	–	–	–	–	–
*p*
Max fails
*R*	−0.743	1	–	–	–	–
*p*	<0.0001***
Number of events						
*R*	−0.2812	0.297	1	–	–	–
*p*	0.0712	0.0563				
Reaction time						
*R*	0.056	−0.142	0.039	1	
*p*	0.724	0.369	0.804			
*Anxiety state*						
*R*	−0.528	0.486	0.173	−0.073	1	–
*p*	0.0003***	0.001**	0.273	0.644		
Heart rate						
*R*	−0.1581	0.265	0.130	−0.173	0.404	1
*P*	0.323	0.089	0.411	0.272	0.008**

Max fails: maximal number of successive fails (Errors + blanks) post-treatment—baseline. Number of events: number of episodes with more than two successive fail post-treatment—baseline. Reaction time: reaction time of correct trials post-treatment—baseline, Anxiety state: State of anxiety post-treatment—baseline. Heart rate: area under the curve of the heart rate response during the complete experiment. *N* = 42 participants. **p < 0.01, ***p < 0.001.

### Psychosocial Stress Affects Frontoparietal Alpha Synchronization During Resting States

**Figure 2:**
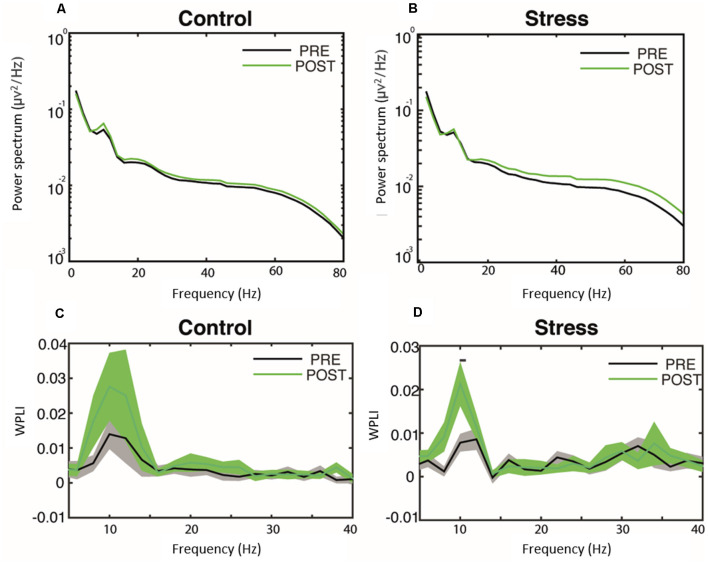
Effect of psychosocial stress over oscillatory activity during resting state. Power spectrum **(A,B)** and phase synchrony through the WPLI **(C,D)** during baseline and after either the control protocol **(A–C)** or the TSST **(B–D)**. The black line over the WPLI spectra in **(D)** indicates *p* < 0.05, permutation test corrected by multiples comparisons. Solid lines and shaded areas illustrate mean and SEM, respectively. The baseline measurement is named ‘pre’. WPLI, Weighted Phase-Lag Index.

Next, we focused on the EEG responses observed during the resting state phase of the TSST and control experiments. We evaluated the average power across channels and frontoparietal phase synchronization changes. Power analysis revealed slight but non-significant differences between the conditions (Figures 2A,B). Conversely, we observed phase-synchronization differences between the frontoparietal electrodes using WPLI analysis. Both control and TSST protocols trigger an increase in alpha synchronization of the frontoparietal electrodes (baseline- vs. post-protocol application comparison). This increase turned significant only after the induction of psychosocial stress (Figure 2D; Line over both curves indicates *p* < 0.05, using a permutation test, corrected by multiple comparisons). The analysis of the control condition failed to be significant due to the high variability observed across subjects during the post-protocol resting state (Figure 2C). We observed no significant differences in the other frequency bands.

### Correct Attentional Trials After the Stress Protocol Are Associated With Increased Beta Power

**Figure 3:**
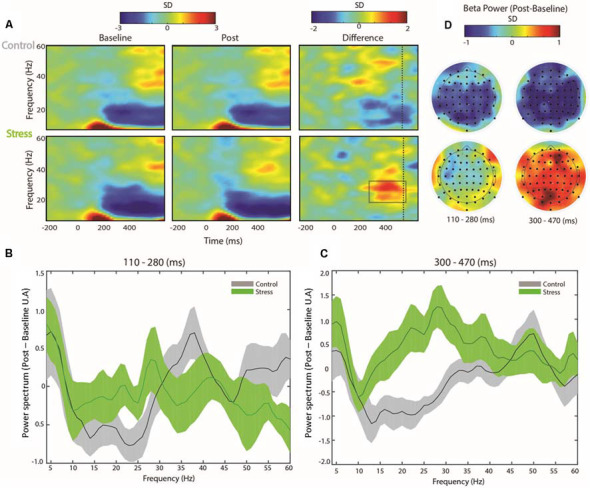
Oscillatory activity associated with correct trials of control and stressed participants. **(A)** Time-frequency charts of correct trials for both groups during baseline and after the control protocol (top) or the TSST (below). The grey square indicates the region in which one difference chart is significantly higher than the other (permutation test corrected by multiple comparisons using a clustering method, Maris and Oostenveld, 2007). The difference chart indicates the spectral power subtracting post spectrograms less baseline. Vertical dashed lines show the mean reaction time. **(B)** Spectrograms of the post-baseline early ( 0.11–0.28 s) and **(C)** late ( 0.3–0.47 s) correct trial differences. **(D)** The topography of the post-treatment beta power (22–28 Hz) less baseline beta power for the control (top) and stress participants (below) during the first (left topographies) and second half (right topographies) of the correct trial.

Beta and gamma rhythms exert top-down and bottom-up modulations in visual cortical areas (van Kerkoerle et al., 2014; Bastos et al., 2015). Therefore, we wondered whether psychosocial stress might impact the neuronal mechanisms underlying the attentional task performance. We observed that the control and stress groups depicted similar time-frequency dynamics during baseline and post trials. Both groups showed an early increase in power at low frequencies, reflecting the evoked potential of the stimulus onset. This early power increase was followed by a decrease in beta-band activity, starting approximately 0.2 s after stimulus onset. We also found a late rise in gamma-band activity (Figure 3A, left middle bottom-up panels) 0.4 s after stimulus onset. Notably, the comparison between the stress and control group time-frequency charts revealed significant differences in the beta frequency band. The psychosocial stress-induced group showed a significant increase of the relative beta-band power between 0.2 and 0.5 s during the attentional task, compared to controls (Figure 3A, bottom-right panel, permutation test, *p* < 0.05, corrected for multiple comparisons). Also, we observed an increase in the gamma-band activity after the control protocol, centered at approximately 0.18 s post-stimulus onset. While this gamma increase was statistically significant after the permutation test, this effect did not survive the cluster method of multiple comparison correction.

We computed the difference between time-frequency charts observed in early (0.11–0.28 s; Figure 3B) and late (0.3–0.47 s; Figure 3C) analysis windows. The early analysis window shows an increase in gamma frequency band power (centered around 35–40 Hz) for the control group. Conversely, the comparison between the stress and control groups in the late analysis window revealed a specific increment of the stress group’s power between 22 and 28 Hz, compared with the control group. We observed this relative beta increase across attentional tasks in the second half of each trial. This effect spread out throughout the scalp, showing higher intensities in the frontotemporal regions (Figure 3D, bottom-right).

Conversely, at earlier latencies, the relative beta increase was almost absent in the stress group and did not show any specific topology (Figure 3D, bottom-left). In the control group, a general decrease in beta was observed at earlier and later latencies. Specific topologies for both latencies were not observed in this group (Figure 3D, top, right, and left). These results suggest that, depending on previous exposure to TSST, different brain rhythms are elicited when participants are engaged in an attentional task.

### Beta Amplitude Power Correlates With Behavioral Performance and the Physiological and Subjective Stress Response

**Figure 4:**
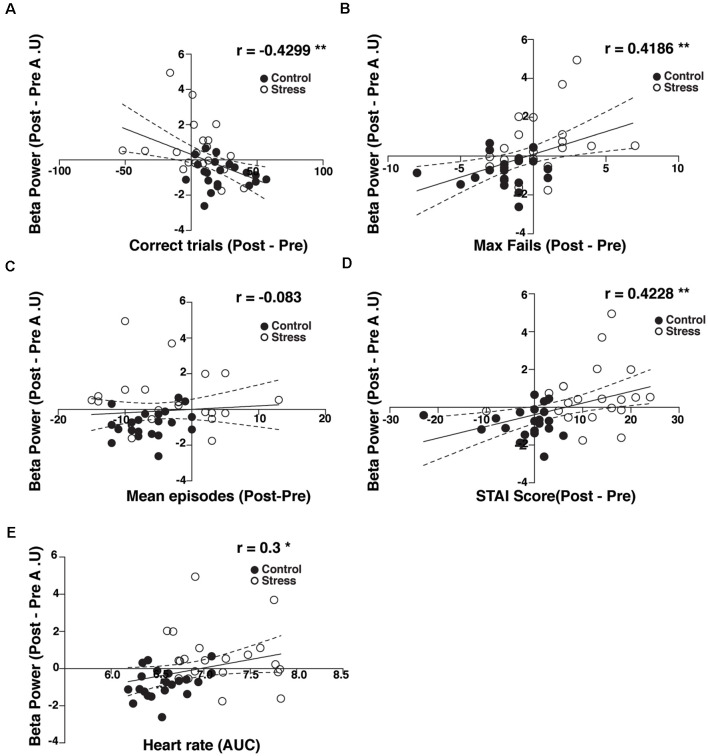
Relationship between beta power, performance, and stress outcomes.** (A)** Person correlation between beta power difference and correct trials. **(B)** The maximal number of consecutive fails. **(C)** The number of episodes with two or more consecutive fails. **(D)** Self-reported state of anxiety. **(E)** Heart rate (area under the curve). *p < 0.05, **p < 0.01.

We quantified the relationship between observed beta activity and attentional performance, anxiety, and HR. We performed a Pearson correlation between beta-band activity and the different measured parameters across all participants for each participant. During the task performance, the beta band power was negatively correlated with the number of correct trials (Figure 4A, *R* = −0.4299, *p* < 0.01), positively correlated with the maximal number of consecutive mistakes (Figure 4B, *R* = 0.4186, *p* < 0.01), and uncorrelated with the number of episodes with two or more consecutive failures (Figure 4C, *R* = −0.083, *p* > 0.05). We also found a positive correlation between beta-band activity, self-reported state of anxiety (Figure 4D, *R* = 0.4228, *p* < 0.01), and HR reactivity (Figure 4E, *R* = 0.3, *p* < 0.05). These correlations suggest that attentional performance is directly related to an increase in power in the beta band, self-reported state of anxiety, and HR changes induced by psychosocial stress.

### Beta Amplitude Power Accurately Predicts Psychosocial Stress Intervention

**Figure 5:**
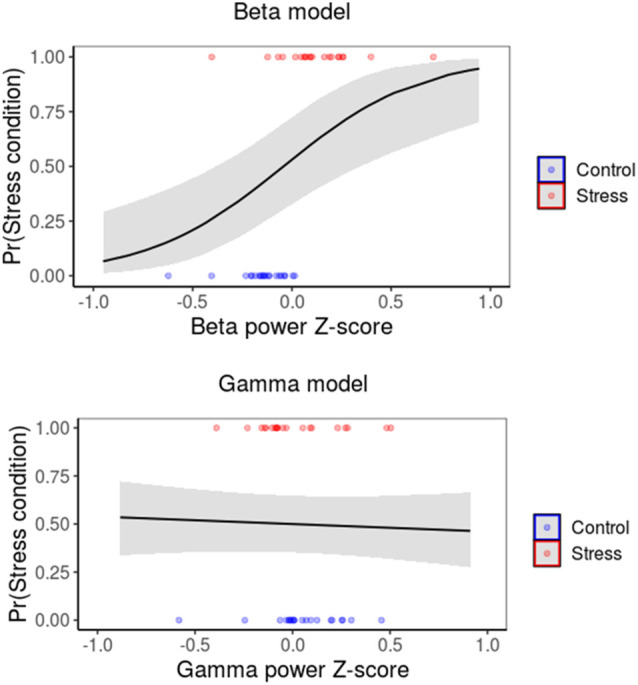
A model including Beta-band and Gama-band power as the covariate. The probability of the stress group occurrence is given by Beta-band or Gamma-band power. The colored points represent the beta or gamma normalized power from each participant performing the task after the TSST or control protocol (red and blue, respectively).

To evaluate whether the abovementioned changes observed in the EEG are informative of the participant’s state during the task, we fitted three different models to the data. We used the intercept as a reference and two covariate models using beta and gamma frequency band power fluctuations (Figure 5). We computed a likelihood ratio test comparing both beta and gamma band additions into the model (see “Materials and Methods” section). Crucially, only beta band addition accounted for information compared to our intercept-only model (beta band addition $\chi_{\left(2\right)}^{2}$ = 23.591, *p* = < 0.001; gamma-band addition $\chi_{\left(2\right)}^{2}$ = 0.2487, *p* = 0.618; Table 2). Wald’s tests revealed that the beta band activity model was significant (Wald’s $\chi_{\left(1\right)}^{2}$ = 10.129, CI [1.1201, 4.7111], *p* = 0.0014) with a positive log-odds ratio (2.9156; Supplementary Table 1), while the gamma band activity model did not reach statistical significance (Wald’s $\chi_{\left(1\right)}^{2}$ = 0.2459, CI [0.7756, 0.4223], *p* = 0.62; Supplementary Table 2). We performed repeated K-fold cross-validations (10-folds, 10 repetitions) to control for the reduced sample size. This measurement also helped us to test the predictive power of the model. The beta model performed with a mean accuracy of 0.85 (Supplementary Table 3; better than the random classifier, Kappa = 0.7044). Finally, we evaluated the model performance by calculating the area under the receiver operating characteristic curve, thus effectively measuring the performance over multiple thresholds. This analysis showed an improvement in the beta model compared to the gamma and intercept models. The poor performance of the gamma model was compared to that of the intercept-only model since all performance metrics were nearly identical (Supplementary Tables 4, 5).

**Table 2:** Logistic regression models.

	Likelihood ratio test			
	Df	Log likelihood	*x*^2^	Pr > *x*^2^
Intercept only	1	−29.112		
Beta model	2	−17.317	23.591	<0.001
Gamma madel	2	−28.998	0.2487	<0.618

## Discussion

The present study evaluated the relationship between psychosocial stress and brain rhythm-specific modulation. Our protocol combined the implementation of a psychosocial stress test with EEG recordings, while experimental subjects performed a cognitive task before and after stress induction. We were able to quantify the behavioral changes that psychosocial stress can induce in a cognitive task and the electrophysiological signatures of such changes. We found that the increase in psychosocial stress and stress-related anxiety was linked to specific changes in beta-band oscillatory activity.

Our findings can be summarized as follows. First, we found a positive correlation between the self-reported state of anxiety and the maximal number of consecutive failures (errors + blanks) in the attentional task. In contrast, we did not find a correlation between the self-reported state of anxiety and the number of episodes during which participants failed consecutively. These two related findings suggest that individuals tend to allocate attentional resources outside the task, independent of anxiety levels. Consequently, as anxiety levels increase, the reallocation of attentional resources back to the task becomes harder (Eysenck et al., 2007). Furthermore, we did not find significant correlations between behavioral performance and physiological activation parameters, but physiological activation correlated positively with the self-reported state of anxiety. This correlation bears out the notion that, at this temporal scale, the physiological responses to stressful experiences may play a role in the psychological perception of stress rather than directly affecting attention (Palacios-Garcia et al., 2017). Second, we conducted electrophysiological recordings at several stages of the experiment. We found a modulation of beta-band power amplitude in participants who underwent the stress-induced protocol. Participants exposed to TSST exhibited a late increase in beta-band activity during the same task. Additionally, we observed a small increase in early gamma-band power in individuals exposed to the control protocol during correct trials in the attentional task. Also, we found a slight increase in frontotemporal alpha phase synchronization in both groups (Figure 2). This comparison turned slightly significant after psychosocial stress induction, probably because of the lower signal-to-noise ratio of our synchronization estimation. Finally, we performed a logistic regression aiming to correlate the observed behavioral changes with the observed electrophysiological results. Using this approach, we found that beta activity significantly predicted the experimental conditions in which the subjects had been tested.

### Stress-Dependent Anxiety Modulates Attentional Processing

The anxiety increase secondary to TSST directly correlated with the maximal number of consecutive mistakes, suggesting that the allocation of attentional resources might shift outside the task during stressful situations (Table 1). This finding corresponds with previous studies that observed a reallocation of attentional resources secondary to incoming emotional or threatening stimuli (van Honk et al., 2001; Roelofs et al., 2007; Ellenbogen et al., 2010), and a general decrease in the behavioral performance induced by psychosocial stress during non-emotional tasks (Vedhara et al., 2000; Plessow et al., 2012; Olver et al., 2014). Additionally, this observation supports the notion that activation of the noradrenergic system generates a shift from flexible, reflexive, focused, top-down processing to an automatic, generic, bottom-up processing (Arnsten, 2009, 2015; Hermans et al., 2011). This type of stress-associated functional response widely impairs executive functions, including abstract processes, such as metacognition and effortful attention (Reyes et al., 2020).

It is worth noting that the attentional task in our experimental design did not provide emotional modulation. However, the TSST protocol triggered several emotional responses, such as augmented HR and anxiety (Figure 1). Therefore, the decrease in behavioral performance during the attentional task can be explained by the emotional experience induced by the TSST protocol. This response might affect the ability to perform appropriately during the attentional task and ultimately decreasing the performance of the subjects (Eysenck et al., 2007). Importantly, the effects of the stress protocol on attentional performance did not correlate with the physiological response (HR), but with the psychological response to stress represented by the increase in anxiety. In our study, the performance of the participants in whom anxiety did not increase was not affected. Usually, the effects of stress on cognitive processes are considered secondary to physiological responses, such as elevated cortisol and sympathetic activation (Vedhara et al., 2000; Elzinga and Roelofs, 2005). However, stress can also affect cognitive functions through mechanisms that are independent of physiological responses (Shields et al., 2016; Ali et al., 2017). Our analyses suggest that the observed psychological response to psychosocial stress was sufficient to modulate attentional processing during task execution and posed the question of what cognitive and neurophysiological mechanisms can reallocate attentional resources during psychosocial stress.

### Psychosocial Stress Direct Attention Internally

We observed an increase in frontoparietal alpha-band synchronization during the resting-state period after stress induction (Figures 2C,D). The increase in alpha frequency band activity has been associated with the inhibition of attention directed toward external events, and the facilitation of internally generated cognition, such as imagery, interoception awareness, and access to memories (Cooper et al., 2003; Klimesch, 2012; Benedek et al., 2014; Villena-González et al., 2017). The experience of stressful situations may prompt subjects toward endogenous cues such as repetitive, intrusive, or self-referential thoughts and strategies of emotional regulation, among others. This orientation toward internal cues might induce an increase in alpha-band activity, decreasing the ability to perform a stimulus-driven externally oriented task (Knyazev et al., 2005; Knyazev, 2013; Tortella-Feliu et al., 2014; Villena-González et al., 2016; Forner-Phillips et al., 2020). In line with this interpretation, previous studies have shown that increases in frontoparietal functional connectivity in the alpha frequency range could reflect difficulties in disengaging from threat-related thoughts in patients with PTSD (Imperatori et al., 2014; Dayan et al., 2016). In our study, we observed a similar increase in the alpha-band phase synchronization for both control and psychosocial induced stress groups, but the difference was only significant for the psychosocial induced stress group. This group exhibited small inter-subject variability compared to the control group. The high variability in the control group can be explained by individual differences in the cognitive state during the resting state of the participants, suggesting that some of them were highly absorbed by thoughts, while others were focused on the external environment.

### Beta Band Increase May Reflect Top-Down Processing

During the attentional task, we observed a decrease in the beta band power after stimulus onset, as described previously (for example, Bosman et al., 2010); however, the comparison between conditions revealed a relative increase in the beta band power of the stress-induced group (Figure 3). Traditionally, the post-stimulus decrease in beta power has been related to the control of motor functions (Baker, 2007). However, in our study, the association between beta power fluctuation and the endogenous modulation of psychosocial stress under the same behavioral outcome argues against this activity as a mere reflection of a motor outcome. In line with our findings, recent studies have shown a more prominent role of beta activity during several sensory and high-level cognitive processes. Some authors have argued that beta oscillations convey moment-to-moment top-down modulatory signals to lower sensory cortices, maintaining the “status quo” of existing mental states (Engel and Fries, 2010; Bressler and Richter, 2015). Under such a framework, a decrease in beta power may reflect a transition towards a stimulus-driven state. Intriguingly, our results show a significant relative increase in beta activity observed in the social stress-induced group, a reflection of the brain activity oriented toward top-down cues.

The increase in beta-band activity after the application of TSST correlated with self-reported anxiety and the maximal number of consecutive mistakes (Figures 4B–D). As such, the relationship between anxiety, task performance, and increased beta band rhythmicity supports the hypothesis of “attentional control,” as proposed by Eysenck et al. (2007). In this hypothesis, the increase in anxiety turns attention out of the ongoing task to the threatening experience, leading to a higher number of consecutive mistakes. In our experiments, stressed individuals could redirect attention to the task, increasing top-down control reflected as an increment of beta frequency-band power.

Furthermore, our logistic regression model (Figure 5) revealed a significant relationship between beta-power modulation and the probability of subserving psychosocial stress. Our model suggests that exposure to stress correlates with a relative increase in beta-power.

In our analysis, we fitted a binary classifier to retain an adequate level of performance, as increasing the granularity of the classification can be associated with a drop in overall model performance (Hou et al., 2015), which was also necessary because the intended purpose of the model was to be highly interpretable, hence, there was no major feature extraction aside from the mean band-power levels. The logistic regression model used in this study provides a parsimonious interpretation of the relative amplitude changes of the beta power, and serves as a good electrophysiological marker of stress and anxiety (Poppelaars et al., 2018), with promising implications on neurofeedback interventions designed to alleviate stress (van Son et al., 2020).

### Clinical Implications and Other Considerations

These results may provide relevant evidence for various clinical considerations. Several studies have reported how different affective styles or personality patterns show differential responses to stress (Silva et al., 2017, 2018). Moreover, stress sensitivity and its regulation are considered to be key aspects in the development of various forms of psychopathology (Doom and Gunnar, 2013; Sloan et al., 2017). In this context, one of the central aspects of the trajectory of the lifespan stress response is how individuals regulate ongoing negative emotions, where cognitive mechanisms of regulation rely heavily on attention (McRae and Gross, 2020). The evidence collected in the present study indicates that attentional mechanisms are redirected to internal aspects (endogenous cues) outside the experimental task, suggesting that this attention shift hinders the correct analysis of the environment and its complexity (task performance). This observation adds to other studies that have shown that stress impairs effortful attention and executive functions. As treatments for different psychopathological disorders often point to attentional mechanisms as the main therapeutic target (Sheppes et al., 2015), it is important to consider our observations as a guide for the development of such strategies. Additionally, as mentioned above, the data obtained point to the beta power band as a relevant candidate for neurofeedback treatments. The combination of cognitive (attentional) therapy strategies and beta band amplitude feedback could offer an excellent treatment tool for stress disorders.

### Limitations of This Study

Our work introduces an electrophysiological setup to the classical TSST procedure to study brain changes caused by psychosocial stress. Nonetheless, we must consider that the attentional task used in this protocol was not designed to assess differences between the top-down and bottom-up attentional processes, which may be essential to consider in further studies using the same setup. Importantly, the 64-channel EEG recording did not allow a more in-depth analysis of the signal source reconstruction. Future experiments with high-density electrophysiological recordings, complemented with hyperscanning or hemodynamic techniques may help to specify an adequate localization of the sources of the effects observed in this study. Also, it is necessary to include multiple measurements after the recovery period to evaluate the effects of socially induced stress over time.

## Conclusions

Our study highlights the complexity of stress response concerning attentional modulation, emotional response, and brain activity. Our findings suggest a possible compensatory strategy allowing stressed participants to self-regulate attentional shifts during stressful experiences. However, this compensation comes at the price of more substantial cognitive and physiological demands. Among the different significant fluctuations of several brain rhythms, the activity of the beta frequency band accurately reflects these attentional changes. The magnitude of the beta power significantly predicts the stress occurrence probability and posits a promising electrophysiological marker of the stress response. Additional studies designed specifically to test this observation should be performed.

While our study shows behavioral and neurophysiological effects of psychosocial stress during the performance of a cognitive task, it is an open question to understand how stress can affect the processing of stimuli with emotional valence (Aldunate et al., 2018), or what the mechanisms are that underly the stress effects over top-down or bottom-up attentional modulations. In summary, our study presents a multilevel perspective for an integrative understanding of the mechanisms underlying psychosocial stress and attentional control. contributing to the task programming.

## Data Availability Statement

The raw data supporting the conclusions of this article will be made available by the authors upon request, without undue reservation.

## Ethics Statement

The studies involving human participants were reviewed and approved by Bioethics Committee of the Faculty of Medicine at the Pontificia Universidad Católica de Chile. The patients/participants provided their written informed consent to participate in this study.

## Author Contributions

IP-G and ER designed the research. IP-G, JS, GC-A, MV-G, CA-V, and ER performed the research. IP-G, ER, NL, and CB analyzed the data. IP-G and CB wrote the manuscript. All authors contributed to the article and approved the submitted version.

## Conflict of Interest

The authors declare that the research was conducted in the absence of any commercial or financial relationships that could be construed as a potential conflict of interest.
