# Supplementary material for: Increase in Beta Power Reflects Attentional Top-Down Modulation After Psychosocial Stress Induction
Source: Front Hum Neurosci. 2021 Mar 23;15:630813. doi: 10.3389/fnhum.2021.630813 (PMC8021732; doi:10.3389/fnhum.2021.630813)

Supplementary Material

# Supplementary Figures and Tables


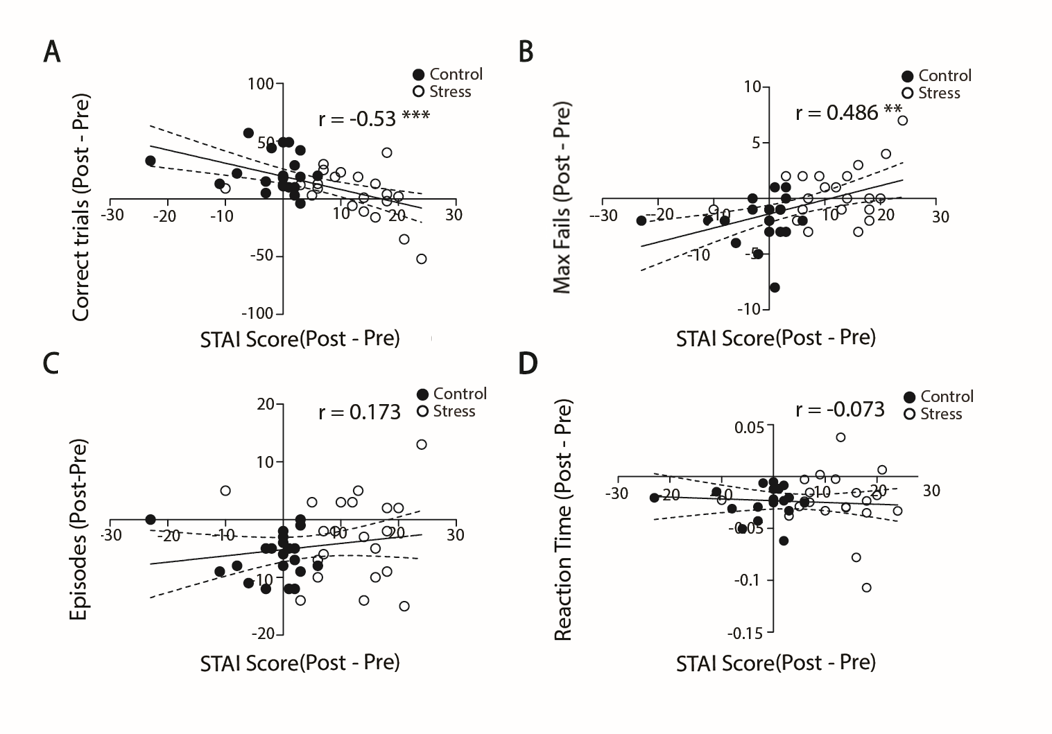


1. **Figure S1ǀ Relationship between anxiety state and performance.** (A) Person correlation between anxiety state difference (Post-Pre) and correct trials. (B) Maximal number of consecutive fails. (C) Number of episodes with 2 or more consecutive fails and (D) Reaction time. **p<0.01, **p<0.001.

**Table S1: Wald test for beta power**


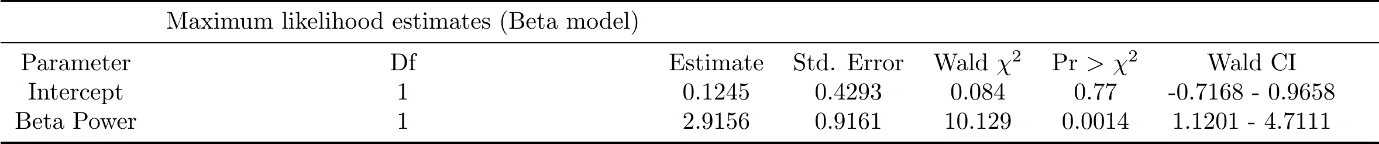


**Table S2: Wald test for gamma power**


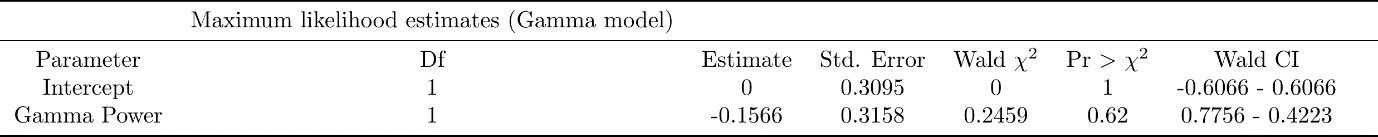


**Table S3: Beta model performance**

**
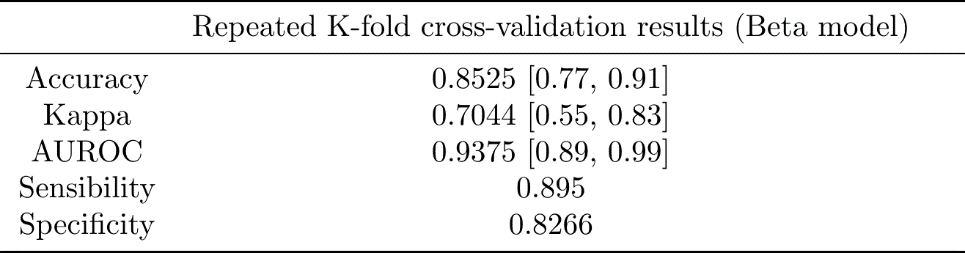
**

**Table S4: Gamma model performance**


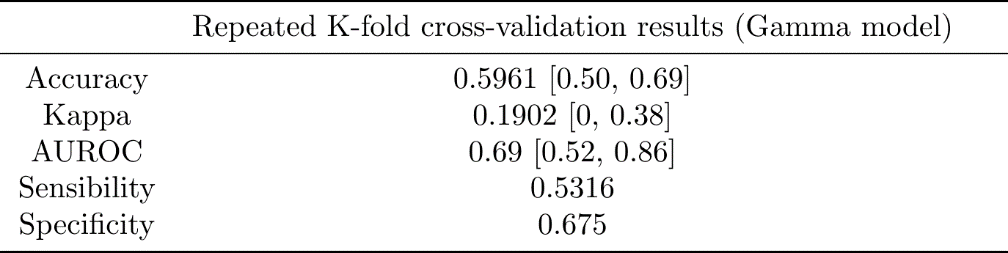


**Table S5: Intercept only performance**


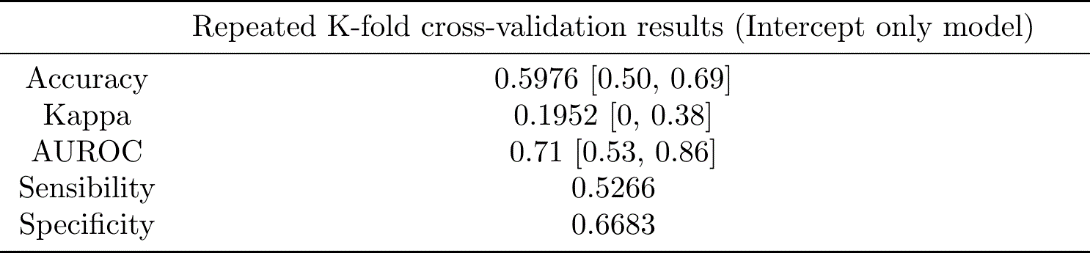

Supplement: Supplementary file 1 [file Table_1.DOCX]
